# Cerebrospinal fluid level of proNGF as potential diagnostic biomarker in patients with frontotemporal dementia

**DOI:** 10.3389/fnagi.2023.1298307

**Published:** 2024-01-25

**Authors:** Francesca Malerba, Rita Florio, Ivan Arisi, Chiara Zecca, Maria Teresa Dell’Abate, Giancarlo Logroscino, Antonino Cattaneo

**Affiliations:** ^1^Fondazione European Brain Research Institute (EBRI) Rita Levi-Montalcini, Rome, Italy; ^2^Institute of Translational Pharmacology – National Research Council (IFT-CNR), Rome, Italy; ^3^Center for Neurodegenerative Diseases and the Aging Brain, Department of Clinical Research in Neurology of the University of Bari “Aldo Moro” at “Pia Fondazione Card G. Panico” Hospital Tricase, Lecce, Italy; ^4^Department of Basic Medical Sciences, Neuroscience and Sense Organs, University of Bari “Aldo Moro”, Bari, Italy; ^5^BIO@SNS Laboratory, Scuola Normale Superiore, Pisa, Italy

**Keywords:** frontotemporal dementia, proNGF, immunoassay, biomarker, diagnosis, neurodegenerative disease, tauopathy, NGF dysmetabolism

## Abstract

**Introduction:**

Frontotemporal dementia (FTD) is an extremely heterogeneous and complex neurodegenerative disease, exhibiting different phenotypes, genetic backgrounds, and pathological states. Due to these characteristics, and to the fact that clinical symptoms overlap with those of other neurodegenerative diseases or psychiatric disorders, the diagnosis based only on the clinical evaluation is very difficult. The currently used biomarkers help in the clinical diagnosis, but are insufficient and do not cover all the clinical needs.

**Methods:**

By the means of a new immunoassay, we have measured and analyzed the proNGF levels in 43 cerebrospinal fluids (CSF) from FTD patients, and compared the results to those obtained in CSF from 84 Alzheimer’s disease (AD), 15 subjective memory complaints (SMC) and 13 control subjects.

**Results:**

A statistically significant difference between proNGF levels in FTD compared to AD, SMC and controls subjects was found. The statistical models reveal that proNGF determination increases the accuracy of FTD diagnosis, if added to the clinically validated CSF biomarkers.

**Discussion:**

These results suggest that proNGF could be included in a panel of biomarkers to improve the FTD diagnosis.

## 1 Introduction

Frontotemporal dementia (FTD) is a heterogeneous and complex neurodegenerative disease, exhibiting different phenotypes, genetic backgrounds, and pathological states. The FTD term encompasses a group of clinical syndromes that are characterized by progressive changes in behavior, executive function, or language (Bang et al., 2015). Although FTD is the second most prevalent early onset dementia, second exclusively to Alzheimer’s disease (AD; Boeve et al., 2022), it is a relatively rare disease (Mercy et al., 2008). The epidemiology of FTD could vary according to a geographical distribution. Surveillance of FTD in the population is difficult and expensive because disease frequency is low. Most of the studies were conducted in Europe or North America, with >95% Caucasian samples (Onyike and Diehl-Schmid, 2013). Two population-based studies in Europe (Coyle-Gilchrist et al., 2016; Logroscino et al., 2019) reported a crude incidence of FTD of 1.6 per 100,000 person-years and 3 per 100,000 person-years, respectively (Zecca et al., 2022).

Frontotemporal dementia occurs both in familial and sporadic forms, with 10%–20% of cases linked to genetic mutations (Boeve et al., 2022). The most common genes linked to familial FTD are: *MAPT* (microtubule associated protein tau), *GRN* (progranulin), and *C9orf72* (chromosome 9 open reading frame 72) (Rohrer et al., 2009; Greaves and Rohrer, 2019; del Campo et al., 2022). Based on clinical presentation, three main syndromes have traditionally been described, namely (a) behavioral variant frontotemporal dementia (bvFTD), with early behavioral and personality changes, (b) non-fluent primary progressive aphasia (nfPPA) with prevalent language impairment in word production, and (c) semantic variant PPA (svPPA), with impairment of semantic knowledge (Boeve et al., 2022; del Campo et al., 2022; Zecca et al., 2022). FTD can also occur with motor neuron pathway involvement, such as FTD with concomitant amyotrophic lateral sclerosis (FTD-ALS) (Strong et al., 2009), or with motor involvement of the extrapyramidal pathway as corticobasal syndrome (CBS) (Armstrong et al., 2013), or progressive supranuclear palsy (PSP) (Litvan et al., 1996).

From the neuropathological point of view, FTD is typically (but not always) associated with focal degeneration of the frontal and temporal cortices, denoted by the term frontotemporal lobar degeneration (FTLD). FTLD involves one or more proteinopathies: 50% of FTD patients have aggregates of TAR DNA-binding protein 43 (TDP-43, FTLD-TDP) while 45% of FTD patients develops aggregates of the protein tau (Irwin et al., 2015). Less than 5% exhibits aggregates of RNA-binding protein fused in sarcoma [FUS (FTLD-FUS)] (Neumann et al., 2009) or ubiquitin-positive inclusion (FTDL-UPS) (Cairns et al., 2007). TDP-43 inclusions are also observed in >95% of ALS patients (Geser et al., 2009; Lee et al., 2017), supporting the hypothesis that FTD and ALS are part of a pathological continuum (Ferrari et al., 2011).

The onset, the first phase and the late stage of the natural history of the pathology are characterized by an overlap of symptoms in different domains: cognition, behavior, language, and movement. Moreover, since the main phenotypes of bvFTD and PPA are mixed, their symptoms and signs are often similar to those observed in AD or in primary psychiatric disorders such as schizophrenia, bipolar affective disorder, and major depression (Velakoulis et al., 2009; Woolley et al., 2011). In the psychological domain, the early and core symptom of FTD, in most of patients, is a deficit in social cognition, which is not readily recognized, and is difficult to measure with objective parameters (Gorno-Tempini et al., 2011; del Campo et al., 2022). It appears evident that a diagnosis based only on clinical evaluation is difficult and can be delayed for up to 6 years (Scialò et al., 2020).

The clinical, pathological, and genetic complexity of FTD requires some efficient biomarkers to increase diagnostic accuracy, identify disease staging and predict, monitor, and measure FTD disease progression (Rosen et al., 2020; Boeve et al., 2022; del Campo et al., 2022). Indeed, the combination of amyloid-β 42 (Aβ42), total tau protein (Tau), and hyperphosphorylated tau 181 (Ptau) in CSF, currently measured for diagnosing biological AD (Jack et al., 2018), are not useful for the diagnosis of FTD, but rather to rule out AD pathology.

It is largely recognized that proNGF, the NGF precursor, could represent a promising diagnostic biomarker for AD (Pentz et al., 2020), for the onset of prodromal AD (Counts et al., 2016) or for other neurodegenerative diseases (Belrose et al., 2014; Xu et al., 2018; Pentz et al., 2021), but almost nothing is known concerning its potential relationship to FTD. The clinical validation of proNGF as biomarker has been hampered so far by the absence of a reliable and scalable proNGF immunoassay (Malerba et al., 2016). We have demonstrated that NGF and proNGF reciprocally interfere in ELISA, making the measurements of both NGF and proNGF unreliable and dependent on the unknown proNGF/NGF ratio. Indeed, in literature NGF and proNGF are often measured in post mortem tissue by low-sensitive immunoblot (Tiveron et al., 2013; Counts et al., 2016; Pentz et al., 2020).

We have recently developed and validated a new assay to measure proNGF in the CSF of living patients (Malerba et al., 2021). This novel method is sensitive, robust, specific, automated and does not suffer the limitation of NGF interference (Malerba et al., 2016). In this article, we have used this immunoassay to measure proNGF in CSF of living patient affected by the FTD spectrum and compared the results to those obtained in CSF from 84 AD, 15 subjective memory complaints (SMC), and 13 control subjects. A statistically significant difference between proNGF levels in FTD compared to AD, SMC and controls subjects was found. Mostly important, the statistical models reveal that proNGF determination increases the accuracy of FTD diagnosis, if added to the set of clinically validated CSF biomarkers. This suggests that proNGF could be included in a panel of biomarkers to improve the FTD diagnosis.

## 2 Material and methods

### 2.1 Patients

Forty-three FTD patients (21 bvFTD, 3 FTD-ALS, 3 PSP, 3 CBS, and 13 PPA, of which 4 patients with nfPPA) were enrolled at the Center for Neurodegenerative Diseases and the Aging Brain of the University of Study of Bari “Aldo Moro” at Pia Fondazione “Card. Panico” Hospital (Tricase). Patients with diagnosis of AD (*n* = 84), SMC (*n* = 15) and control subjects (CTR) (*n* = 13), enrolled in the same clinical center by the same medical team and previously described (Malerba et al., 2021) were also considered.

The study was approved by the Local Ethical Committee, according to the Helsinki Declaration. All study participants gave written informed consent, and a structured interview exploring familiar, personal and medical history, social status, and overall physical exam was performed (Maggi et al., 1994). Each subject underwent a multidisciplinary assessment with a neurological and neuropsychological examination, a MRI-3T scan and a routine laboratory assessment. The FrontoTemporal Lobar Degeneration – Clinical Dementia Rating Scale Sum of Boxes (FTLD-CDR SOB) (Miyagawa et al., 2020) was the tool to assess the severity of the disease. General cognition was assessed by the Mini-Mental State Examination (MMSE). Other variables included were the age at the first symptom and the age at the date of lumbar puncture. The disease duration (in months) was calculated as the difference between the date of the diagnosis and the date of the first symptom. Demographic data including age, sex, and years of education were collected. The fulfillment of the current clinical criteria of the FTLD spectrum (Strong et al., 2009; Gorno-Tempini et al., 2011; Rascovsky et al., 2011; Armstrong et al., 2013; Höglinger et al., 2017) and the absence of significant medical or psychiatric illness, such as major depression, schizophrenia, or bipolar disorders were inclusion criteria.

Patients with AD (*n* = 84) were diagnosed using standard diagnostic criteria for dementia [Diagnostic and Statistical Manual of Mental Disorders, 5th ed. (DSM-5)] (American Psychiatric Association, 2013) and National Institute on Aging-Alzheimer’s Association (NIA-AA) criteria (McKhann et al., 2011). Patients included in this group had CDR score equal 1 and the MMSE score <24.

Standard cut-off points of MMSE were also used to classify dementia severity into mild (MMSE ≥ 20), moderate (10 ≤ MMSE ≤ 19), and severe stages (MMSE ≤ 9) (Folstein et al., 1975). When cut-off values on the MMSE were applied to our cohort patients, 30 (36%) patients had mild AD dementia, 42 (50%) had moderate AD dementia, and 12 (14%) had severe AD dementia.

This staging was applied also to the FTD cohort, although the clinical diagnostic criteria for FTD include relative preservation of memory and visuospatial function, in contradistinction to characteristics of AD. Based on the MMSE cut-off, 23 (66%) patients had mild dementia, 12 (34%) had moderate dementia, none presented severe dementia.

A total of 80% (n. 67) of the AD patients were taking anticholinesterases, the approved symptomatic therapies for AD, known to improve memory by increasing brain acetylcholine levels. On the contrary, the use of these agents in FTD have shown disappointing results, so routine use is not recommended (Tsai and Boxer, 2016).

Patients diagnosed with SMC presented subjective memory concern and were “self-referrals.” Criteria for diagnosis were: (1) self-experienced persistent decline in memory and cognitive capacity in comparison with a previously normal status and unrelated to an acute event; (2) normal age-, gender-, and education-adjusted performance on standardized cognitive tests, which are used to classify mild cognitive impairment (MCI) or prodromal AD (Jessen et al., 2014). CDR = 0 and MMSE score between 24 and 30 (inclusive) were considered for this group.

According to ADNI-3 criteria (Weiner et al., 2017), a group of patients was considered as control group. These subjects were patients spontaneously gone to the clinical center, for a subjective cognitive decline. These patients underwent the entire clinical assessment foreseen by the center, including the lumbar puncture. At the end of the diagnostic process they showed:

– absence of significant impairment in cognitive functions from neuropsychological test performance or daily functioning abilities;
– no signs of depression, mild cognitive impairment, dementia or any significant neurologic disease (Parkinson’s disease, multi-infarct dementia, Huntington’s disease, normal pressure hydrocephalus, brain tumor, PSP, seizure disorder, subdural hematoma, and multiple sclerosis);
– no laboratory alterations (CSF beta-Amyloid42, tau, and pTau);
– imaging MRI scan with no evidence of infection, infarction, lesions with more than 1.5 cm or other focal lesions.

Other specific inclusion criteria were: absence of major depression, bipolar disorder as described in DSM-IV within the past 1 year; no history of schizophrenia (DSM IV criteria); no history of significant head trauma followed by persistent neurologic defaults or known structural brain abnormalities; no history of alcohol or substance abuse or dependence within the past 2 years (DSM IV criteria); Geriatric Depression Scale less than 6; no any significant systemic illness or unstable medical condition; no clinically significant abnormalities in B12, homocysteine (HC) and methylmalonic acid (MMA); no current use of specific psychoactive medications (e.g., certain antidepressants, neuroleptics, chronic anxiolytics or sedative hypnotics, etc.); no current use of warfarin (exclusionary for lumbar puncture).

For all the patients, exclusion criteria at the time of enrollment were considered: therapy with chemotherapies; therapy with antidepressant (i.e., SSRIs); use of antibiotics or anti-inflammatory drugs over the last month; drug or alcohol addiction. Systemic processes such as tumor, including prostate cancer, and severe chronic cardiovascular diseases, were excluded. Patients with other comorbidities were not excluded because in elderly patients, with an average age ranging from 64 to 74 years, multimorbidities are very common. The aim of the project is to validate proNGF as biomarker in real world patients.

As part of the diagnostic procedure, the lumbar puncture for CSF biomarkers analysis (Aβ42, Tau, and pTau), was performed. CSF was obtained by lumbar puncture between the L3/L4 or L4/L5 intervertebral space, and 8 ml was collected in polypropylene tubes. All patients underwent the lumbar puncture in the morning after an overnight fast and after signing a specific written informed consent. Within 1 h, the CSF samples were centrifuged at room temperature for 10 min at 2000 *g* (rcf), aliquoted and stored at −80°C until analysis, according to international biomarkers recommendations (Vanderstichele et al., 2012). The CSF Aβ42, Tau, and p-Tau181 levels were measured by chemiluminescent immunoassay CLEIA (Lumipulse G ß-amyloid 1–42, Lumipulse G Total Tau, Lumipulse G pTau181, Fujirebio Europe N.V., Gent, Belgium) on fully automatic platform (Lumipulse G600II, Fujirebio Europe N.V., Gent, Belgium). All the assays were performed according to manufacturer’s protocols. For the interpretation of the cerebrospinal biomarker results, the following cut-off values, provided by the manufacturer, were considered: Aβ42 >599 pg/ml, Tau <342 pg/ml, and p-Tau181 <57.6 pg/ml.

### 2.2 ProNGF immunoassay

The proNGF immunoassay was carried out with Simple Wes (Protein Simple) as described in Malerba et al. (2021).

Briefly, the calibration curve was carried out by serial dilution of human recombinant proNGF produced in our lab (Malerba et al., 2015), from 4 to 31 ng/ml in 0.1% Wes Sample Buffer (Bio-Techne). The proNGF dilutions were added with 1/5 of Fluorescent Master Mix (Bio-Techne) and then boiled. A total of 130 μl of CSF were desalted by Zeba Spin Desalting Columns (7K MWCO Thermoscientific). The proteins in the sample were precipitated with 20% TCA. Protein pellet was resuspended in 10 μl of 0.1% Wes Sample Buffer, added with 1/5 of Fluorescent Master Mix (Bio-Techne) and then boiled. Calibrators and samples were run in duplicates on Simple Wes (Bio-Techne) by using 2–40 KDa cartridges. Anti NGF MyBioSource (MBS125020) and the Biotin-SP AffiniPure Goat Anti-Rabbit IgG (111-065-003 Jackson Immunoresearch) were used as primary and secondary antibodies, respectively.

As previously mentioned, the samples were denatured, so the resulting molecular weights (MW) corresponded to those of the monomer of the neurotrophins, deduced from the amino acid sequence. The observed slight peak shift with respect to the theoretical MW is due to the strong cationic charge of the neurotrophins and to Simple Wes characteristics. Indeed the Simple Wes does not provide an absolute MW but an apparent one, as reported in the manufacturer’s instructions.

### 2.3 Clinical sample measurement

Forty-three CSF from FTD patients were measured by the proNGF immunoassay. Each sample was tested at least four times, in two different assays. For each sample, mean, standard deviation, and coefficient of variation (CV) were calculated. A CV ≤20% was considered acceptable. In case of CV >20%, the measurements were repeated. For each peak, the area under the curve was computed by the supplied software Compass (Bio-Techne), and statistically analyzed. A signal to noise ratio ≥10.0 was considered acceptable.

### 2.4 Statistical analysis

The Kruskal–Wallis test was used for single factor analysis to compare biomarker levels between the diagnostic groups (FTD, AD, SMC, and controls). Pairwise Mann–Withney test, with multiple testing *p*-value correction, was used as *post-hoc* test. Contingency tables and proportions were analyzed with the Fisher’s exact test. Correlation between biomarkers was quantified by Spearman’s index. Variances between >2 groups were compared by the Bartlett’s test of homogeneity of variances, followed by pairwise *F*-test with multiple testing between groups.

Diagnostic performance of biomarkers was analyzed by logistic regression multivariate models and ROC curves. Optimal cut-offs were identified by the maximum Youden index in ROC curves. For NGF, when the protein was found below the experimental detection threshold, its value was set to zero and included in regressions, but excluded from the analysis for Spearman’s correlation.

We defined four discrete multivariate diagnostic models to discriminate FTD vs. Controls (pool of SMC and CTR subjects) in the following way, including and not including the proNGF and NGF as covariates:


$$\mathrm{Diagnosis}\,\mathrm{score}=\text{Sum}\,\left(A\beta 42_{\text{bin}}+{\text{Tau}}_{\text{bin}}+{\text{pTau}}_{\text{bin}}\right)$$

$$\mathrm{Diagnosis}\,\mathrm{score}=\text{Sum}\,\left(A\beta 42_{\text{bin}}+{\text{Tau}}_{\text{bin}}+{\text{pTau}}_{\text{bin}}\,+{\text{proNGF}}_{\text{bin}}\right)$$

$$\mathrm{Diagnosis}\,\mathrm{score}=\text{Sum}\,\left(A\beta 42_{\text{bin}}+{\text{Tau}}_{\text{bin}}+{\text{pTau}}_{\text{bin}}+{\text{NGF}}_{\text{bin}}\right)$$

$$\mathrm{Diagnosis}\,\mathrm{score}=\text{Sum}\,\left(A\beta 42_{\text{bin}}+{\text{Tau}}_{\text{bin}}+{\text{pTau}}_{\text{bin}}\,+{\text{proNGF}}_{\text{bin}}+{\text{NGF}}_{\text{bin}}\right)$$

where Aβ42_bin_, Tau_bin_, pTau_bin_, proNGF_bin_, and NGF_bin_ are binary variables set = 1.0 if the corresponding Aβ42, Tau, pTau, proNGF, and NGF values fall in the pathological range according to the identified cut-offs, or = 0.0 if they are out of the disease range. Diagnostic score can take only integer values = {0,1,2,3,5}.

In this case, the decision to pool controls and SMC was based on the fact that they show a similar variability, non-significantly different amount of proNGF, and on careful clinical evaluations: both, SMC and controls, were resulted cognitively normal after the clinical assessment, without laboratory alterations. This choice was made in order to have a more robust sample size.

The ROC curves were compared by the one-sided De Long *Z*-test. All statistical analyses were performed using R-Bioconductor, including pROC and ggplot2 packages (Robin et al., 2011; R Core Team, 2013; Villanueva et al., 2016).

Significance threshold for *p*-value was set to 0.05 in hypothesis testing. Benjamini–Hochberg FDR procedure was used for multiple testing *p*-value correction.

## 3 Results

### 3.1 Determination of proNGF level in human CSF samples

Forty-three CSF samples from living FTD patients were measured and analyzed, by using the proNGF immunoassay described in Malerba et al. (2021). The demographic information of patients is listed in Table 1.

**TABLE 1:** Sociodemographic and biomarkers details of the study population.

	FTD	bvFTD	PPA	FTD-ALS	PSP	CBS
*n* (Male + Female)	22 + 21	14 + 7	5 + 8	2 + 1	2 + 1	0 + 3
Age (years) (mean ± SD)	66.7 ± 9.4	64.4 ± 10.0	66.9 ± 9.2	71.6 ± 4.7	74.3 ± 10.2	69.7 ± 4.0
Aβ42 (pg/ml) (mean ± SD)	911.0 ± 321.3	947.1 ± 280.0	796.7 ± 260.8	1,057.3 ± 609.2	855.0 ± 103.1	1,013.3 ± 666.0
Tau (pg/ml) (mean ± SD)	455.5 ± 358.3	328.6 ± 194.5	739.2 ± 528.4	476.3 ± 40.2	271.7 ± 176.2	414.7 ± 128.9
pTau (pg/ml) (mean ± SD)	60.3 ± 43.5	48.2 ± 26.8	91.3 ± 65.1	54.03 ± 8.3	37.6 ± 16.5	54.5 ± 17.7

Values describe the whole FTD population (left) and the five clinical FTD subgroups. Statistical analysis of Tau, Aβ_42_ and pTau in the CSF samples is reported in Supplementary Figure 1.

**FIGURE 1:**
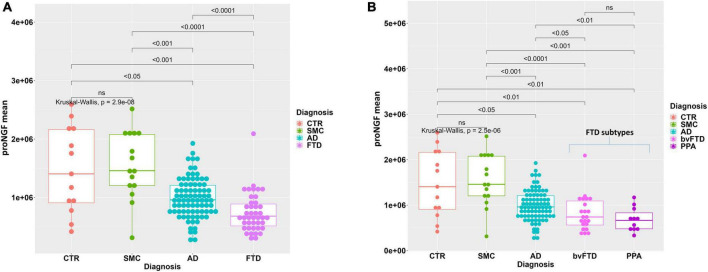
Statistical analysis of proNGF in the CSF samples. **(A)** Boxplot with dotplot: measure of proNGF (peak area) in the diagnostic groups. The outlier in FTD group is a female patient with bvFTD, with no pathological or clinical features that differentiate her from the other patients in the diagnostic group. **(B)** Boxplot with dotplot: measure of proNGF (peak area) in the two main FTD diagnostic subgroups, compared to AD and controls. The boxes enclose data in the median ± IQR range. Kruskal–Wallis test is followed by pairwise Mann–Whitney test with FDR *p*-value correction (horizontal bars). The difference in data dispersion between groups was analyzed by the omnibus Bartlett’s test followed by pairwise variance *F*-test with FDR *p*-value correction.

The current proNGF immunoassay measures the proNGF peak corresponding to the “naked” form of proNGF (MW 34 KDa), without any post translational modification (see Malerba et al., 2021 for details). The areas corresponding to the 34 kDa proNGF peak were therefore analyzed in the FTD CSF samples (Table 2 and Figure 1). The concentrations of proNGF, obtained by interpolating on the calibration curve the median and the average values of the areas of each analyzed FTD subgroup (bvFTD, PPA, FTD-ALS, PSP, and CBS), are listed in Table 2, which also reports the corresponding values for the AD, SMC, and CTR groups.

**TABLE 2:** Mean and median of proNGF peaks area and proNGF concentration for the diagnostic groups (including AD, SMC, and CTR, published in Malerba et al., 2021).

	proNGF mean				proNGF median			
			**Concentration**				**Concentration**	
**Diagnosis**	**area**	**CV (%)**	**(ng/ml)**	**(nM)**	**area**	**CV (%)**	**(ng/ml)**	**(nM)**
AD	990902	11.0	502.9	20.1	960578	10.9	487.5	19.5
FTD	742103	12.8	378.8	15.2	681893	12.3	349.5	14.0
CTR	1475921	11.2	759.4	30.4	1404631	9.6	720.4	28.8
SMC	1554784	9.5	803.3	32.1	1458425	6.4	750.0	30.0
bvFTD	827223	14.1	420.7	16.8	739349	13.2	377.5	15.1
PPA	681146	9.9	349.1	14.0	664416	8.3	341.0	13.6
FTD-ALS	763648	17.6	389.4	15.6	818217	18.6	416.3	16.7
CBS	501748	9.2	263.1	10.5	531878	9.2	277.4	11.1
PSP	560206	13.2	271.9	10.9	689962	13.8	353.4	14.1

The difference between mean and median values in Table 2 suggests that some data distributions are skewed, particularly proNGF for whole FTD and bvFTD diagnostic groups (skewness = 1.61, 1.44, respectively), as also assessed by the Shapiro–Wilk normality test (*p* = 2.84E−04, *p* = 7.08E−03, respectively) and by the D’Agostino test for skewness (*p* = 1.46E−04, *p* = 4.20E−03, respectively). For these reasons we used non-parametric statistical tests to compare diagnostic groups in a one-way factorial design: Kruskal–Wallis test followed by pairwise FDR-corrected Mann–Whitney *post-hoc* tests.

In Figure 1A, the individual areas of the proNGF 34 kDa peaks for each sample of the FTD patients are reported, and compared to the values for the AD, SMC, and CTR groups: there is a significant difference between the FTD group and AD, SMC, and CTR groups (Kruskal–Wallis test *p* = 3.9E−08; *post-hoc* pairwise M-W test FDR <0.001). It is noteworthy that the FTD values are significantly lower than those of the AD group, previously reported to be much lower than the SMC and CTR groups (Malerba et al., 2021). Moreover, the data dispersion in FTD and AD groups is significantly smaller than in SMC and CTR groups, as assessed by the variance test (Bartlett’s test *p* = 1.4E−05; *post-hoc* pairwise Fisher’s test FDR <0.01).

We have then compared the proNGF-34 kDa peak area also between the two main FTD subgroups (bvFTD and PPA) and the AD, SMC, and CTR groups (Figure 1B). A statistically significant difference is evident between either the bvFTD or the PPA subgroups, with respect to AD, SMC, and controls, while no significant difference is observed between bvFTD and PPA.

A higher variability of proNGF in the CSF samples of SMC and CTR groups with respect to the other diagnostic groups is observed. This can be explained by the higher heterogeneity of the control subjects. Moreover, the variability is more evident also due to the smaller sample size with respect to the AD and FTD groups.

### 3.2 Comparison of proNGF CSF levels with clinically validated CSF biomarkers

We assessed the levels of clinically validated biomarkers in the set of FTD CSF samples, alongside the proNGF-34 KDa values and compared them to the corresponding values for the AD, SMC, and CTR groups.

With this set of data, we tested the performance of diagnostic models with single predictors. First, we analyzed each single clinically validated biomarker (Aβ42, Tau, and pTau), which are considered predictive in distinguishing AD from FTD or FTD from controls, separately from proNGF. By binary univariate models, we compared FTD and AD, FTD and SMC_CTR (for this comparison we pooled CTR and SMC subjects). This analysis allowed to find optimal cut-offs for diagnosis by the Youden criterion (maximum index) in ROC curves.

**FIGURE 2:**
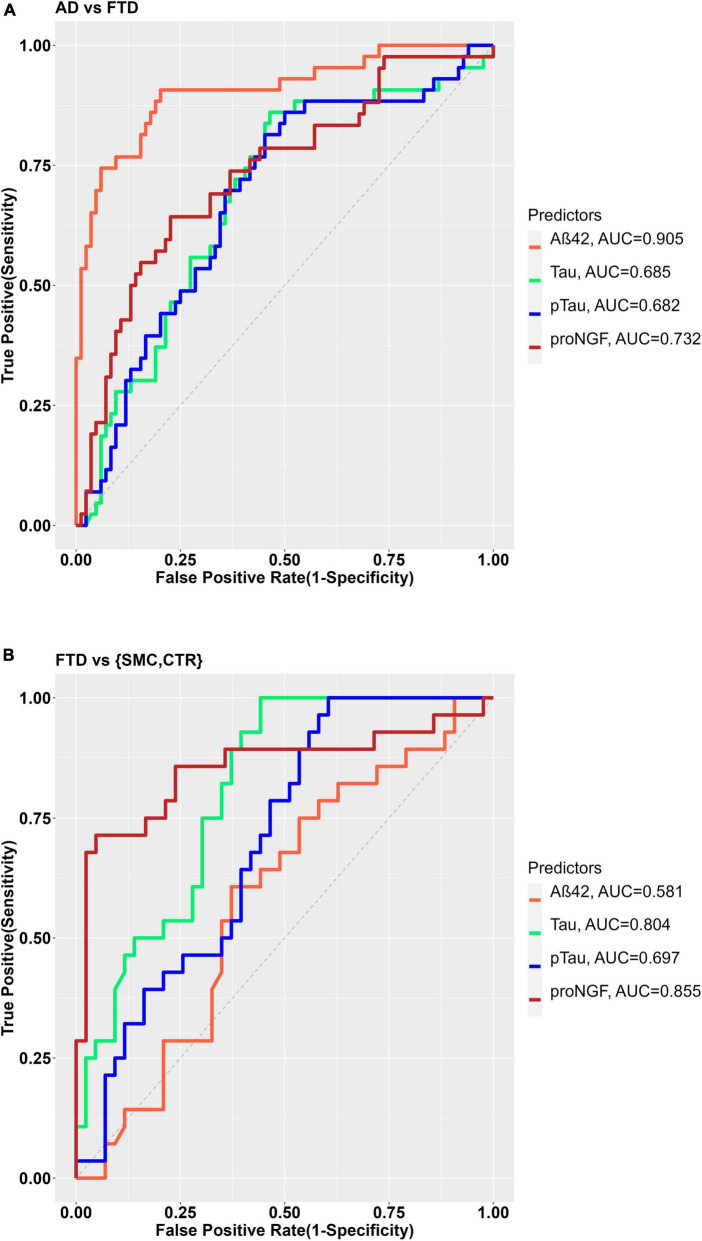
**(A)** ROC curves for the binary diagnostic prediction (AD vs. FTD), using the Aß42, Tau, pTau, and proNGF biomarker values. **(B)** ROC curve for the binary diagnostic prediction (FTD vs. controls), using the Aß42, Tau, pTau, and proNGF biomarker values. The optimal cut-off was estimated by the maximum Youden index.

As expected, the best performance in discriminating AD from FTD is obtained by CSF Aβ42 (Figure 2A), as evident from the largest area under the curve (AUC) of the corresponding ROC curve (AUC = 0.905). The AUC for Tau and pTau are smaller, 0.685 and 0.682, respectively, while the proNGF AUC is 0.732 (Figure 2A), suggesting that proNGF performance in discriminating AD from FTD is better than Tau and pTau.

As for the discrimination between FTD and SMC_CTR, the single biomarker diagnostic models shows that though Tau and pTau levels are able to discriminate FTD from controls with sufficient accuracy (AUC Tau = 0.804; AUC pTau = 0.697), Aβ42 (AUC 0.581) does not discriminate at all (Figure 2B), as expected. Remarkably, in the analyzed groups, proNGF appears as the best biomarker in discriminating FTD from control (AUC = 0.855, Figure 2B).

Moreover, we compared multivariate models using the set of clinically validated biomarkers (Aβ42, Tau, and pTau) as covariates predicting the disease diagnosis, to the same models with the contribution of proNGF as an additional covariate, for the comparison between FTD and AD, and between FTD and SMC_CTR (we pooled CTR and SMC subjects).

**FIGURE 3:**
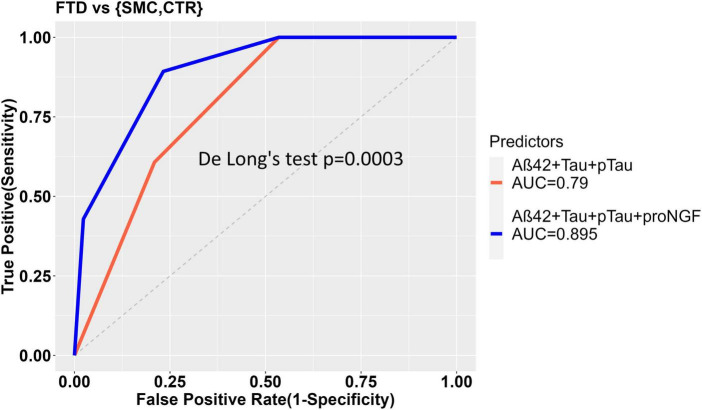
ROC curves to show the diagnostic performance of multivariate models comparing FTD vs. Control samples. The model without proNGF (diagnosis ∼ Aß42 + Tau + pTau), (in red), is compared to the same model with proNGF as further predictor (diagnosis ∼ Aß42 + Tau + pTau + proNGF) (in blue). The two curves are compared using De Long’s test (*p* = 0.0003). The details of the models are described in section “2 Material and methods.”

In the comparison between FTD and SMC_CTR, the ability of Aβ42, Tau, and pTau to predict the diagnostic groups shows a significant improvement when proNGF is included as predictor in the diagnostic model, as witnessed by the significant difference between the two ROC curves (De Long’s test, *p* = 0.00032) and by the increased AUC (AUC = 0.790 for diagnostic model ∼ Aβ42 + Tau + pTau; vs. AUC = 0.895 for diagnostic model ∼ Aβ42 + Tau + pTau + proNGF) (Figure 3).

On the contrary, in the comparison AD vs. FTD, the inclusion of proNGF as predictor does not improve the diagnostic performance (AUC = 0.863 for diagnostic model ∼ Aβ42 + Tau + pTau; vs. AUC = 0.761 for diagnostic model ∼ Aβ42 + Tau + pTau + proNGF) (Supplementary Figure 2).

### 3.3 Detection of mature NGF in human CSF samples

As previously reported in our analysis of AD CSF samples (Malerba et al., 2021), also in a sizeable proportion of the FTD CSF samples, a peak corresponding to mature NGF, at an apparent MW between 16 and 21 KDa, could be identified. Only the NGF peaks having a signal to noise ≥10 were considered acceptable (see section “2 Material and methods”) and analyzed (24/43 FTD samples). The fact that only in some (and not all) of the analyzed samples, mature NGF is detectable is not surprising, because it was reported that the concentration of mature NGF in the brain is significantly lower with respect to that of proNGF (Fahnestock et al., 2001; Counts and Mufson, 2005).

In Table 3 the median and the average values of the NGF peak areas for the whole FTD group and of each FTD subgroup, together with those of AD, SMC and controls are listed. The reported peak areas lack the corresponding concentration values due to the absence of a validated calibration curve for NGF, as previously reported (Malerba et al., 2021).

**TABLE 3:** Mean and median of the NGF peaks area for the diagnostic groups (including AD, SMC, and CTR, published in Malerba et al., 2021).

	NGF mean		NGF median	
**Diagnosis**	**area**	**CV (%)**	**area**	**CV (%)**
AD	261000	16.8	230107	15.8
FTD	277412	17.9	250724	16.4
CTR	379202	12.0	371951	12.2
SMC	381741	12.6	374729	11.3
bvFTD	296552	20.5	280695	22.2
PPA	259844	12.0	214578	11.1
FTD-ALS	262259	32.0	262259	32.0
CBS	219281	10.5	219281	10.5
PSP	246274	19.0	246274	19.0

The difference between mean and median values in Table 3 suggests that the data distribution of NGF for AD is skewed (skewness = 2.08), as also assessed by the Shapiro–Wilk normality test (*p* = 1.97E−03) and by the D’Agostino test for skewness (*p* = 7.74E−04). For these reasons we used the Kruskal–Wallis test followed by pairwise FDR-corrected Mann–Whitney *post-hoc* tests to compare diagnostic groups.

Table 4 reports the number of CSF samples from the various groups, in which the NGF peak was above the predefined threshold for a reliable measure. Inspection of Table 4 clearly shows that the percentage of samples having a detectable NGF peak is significantly higher in FTD, SMC, and CTR than in the AD group (proportions test, *p* < 0.0001), with the SMC and CTR diagnostic groups exhibiting the highest percentage of detectable NGF peak.

**TABLE 4:** Number and percentage of samples having a detectable NGF peak (signal/noise ≥10) for the diagnostic groups.

	Number of samples measured	Number of samples with detectable NGF peak	Percentage (%)	Comparison of proportion vs. AD, proportions test *p*-value
AD	84	13	11	
FTD	43	24	56	*p* < 0.00001
CTR	13	9	69	*p* < 0.0001
SMC	15	11	73	*p* < 0.00001

Percentage in FTD, CTR, and SMC groups are compared to AD by the two proportions *Z*-test.

**FIGURE 4:**
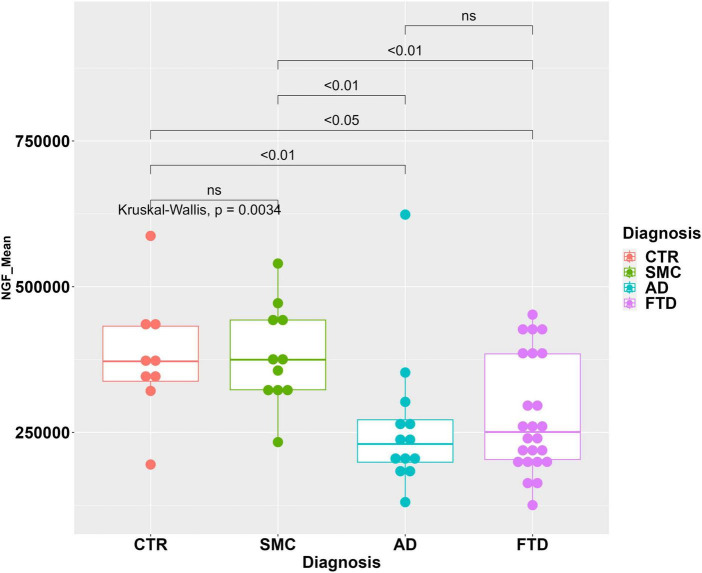
Statistical analysis of NGF in the CSF samples. Boxplot with dotplot: measure of NGF (peak area) in the diagnostic groups FTD, AD, SMC, and controls. The boxes enclose data in the median ± IQR range. Kruskal–Wallis test is followed by pairwise Mann–Whitney test with FDR *p*-value correction (horizontal bars). The difference in data dispersion between groups was analyzed by the omnibus Bartlett’s test followed by pairwise variance *F*-test with FDR *p*-value correction.

The areas of NGF peaks, from the samples in which they could be reliably measured, are reported as boxplots in Figure 4. The NGF levels are significantly lower in FTD samples compared to both CTR and SMC subjects, similarly to the proNGF levels. On the other hand, while the proNGF values in FTD are significantly lower than those in AD CSF samples (see Figure 1), the NGF levels in FTD samples are not significantly different to those in AD (Figure 4).

Despite the method was not fully validated for the NGF peak, unlike the proNGF one (see validation in Malerba et al., 2021), we compared multivariate models using the set of clinically validated biomarkers (Aβ42, Tau, and pTau) as covariates predicting the disease diagnosis, to the same models with the contribution of NGF or NGF and proNGF as additional covariates, for the comparison between FTD and AD, FTD and SMC_CTR (we again pooled CTR and SMC subjects). In the samples in which the NGF peak was absent or with a signal to noise ratio <10, the NGF area was set = 0.

**FIGURE 5:**
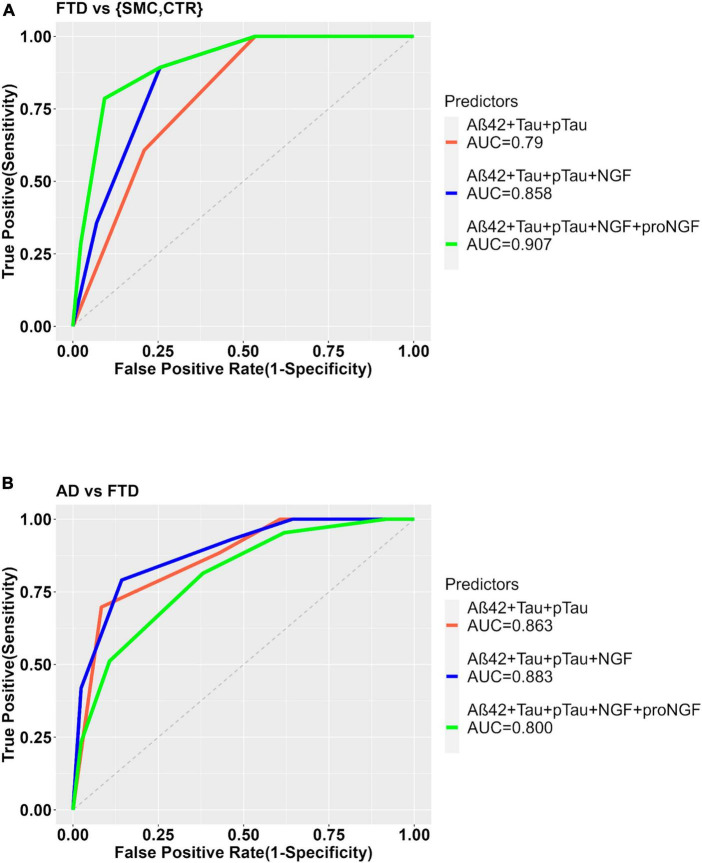
ROC curves to show the diagnostic performance of multivariate models comparing FTD vs. Control samples **(A)** or FTD vs. AD **(B)**. The model with the clinically validated biomarkers (diagnosis ∼ Aß42 + Tau + pTau) is compared to the same model plus NGF (diagnosis ∼ Aß42 + Tau + pTau + NGF) or with proNGF (diagnosis ∼ Aß42 + Tau + pTau + NGF + proNGF) as further predictors.

In the comparison between FTD and the pooled controls, the ability of Aβ42, Tau, and pTau to predict the diagnostic group shows a significant improvement when NGF alone, or NGF + proNGF are included as predictors, as evident from the significant difference between the ROC curves (Figures 3, 5A) and by the increased AUC (AUC = 0.790 for diagnostic model ∼ Aβ42 + Tau + pTau; AUC = 0.858 for diagnostic model ∼ Aβ42 + Tau + pTau + NGF; De Long’s test, *p* = 0.0183); (AUC = 0.790 for diagnostic model ∼ Aβ42 + Tau + pTau; AUC = 0.907 for diagnostic model ∼ Aβ42 + Tau + pTau + proNGF + NGF; De Long’s test, *p* = 0.0032).

In the comparison AD vs. FTD, the inclusion of NGF or NGF + proNGF as predictors does not change the diagnostic performance, as evident from the ROC curves (see Supplementary Figure 2 and Figure 5B) and AUC values (AUC = 0.863 for diagnostic model ∼ Aβ42 + Tau + pTau; AUC = 0.883 for diagnostic model ∼ Aβ42 + Tau + pTau + NGF; AUC = 0.800 for diagnostic model ∼ Aβ42 + Tau + pTau + proNGF + NGF).

**FIGURE 6:**
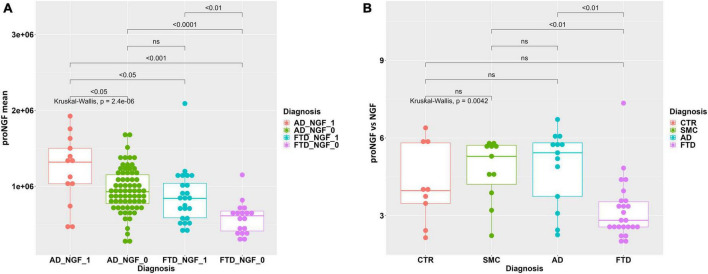
**(A)** Statistical analysis of proNGF in the CSF samples, stratified by NGF detection. Boxplot with dotplot: measure of proNGF (peak area) in AD and FTD groups, stratified according to the presence (_1) or non-presence (_0) of detectable NGF peak. The boxes enclose data in the median ± IQR range. Kruskal–Wallis test is followed by pairwise Mann–Whitney test with FDR *p*-value correction (horizontal bars). **(B)** Statistical analysis of ratio proNGF/NGF in the CSF samples. Boxplot with dotplot: ratio proNGF/NGF in the diagnostic groups FTD, AD, SMC, and controls. The boxes enclose data in the median ± IQR range. Kruskal–Wallis test is followed by pairwise Mann–Whitney test with FDR *p*-value correction (horizontal bars). The difference in data dispersion between groups was analyzed by the omnibus Bartlett’s test followed by pairwise variance *F*-test with FDR *p*-value correction.

When FTD and AD samples are stratified based on the presence/non-presence of a detectable NGF peak (see Table 4), we find a significant difference in average proNGF level between the two subpopulations FTD_NGF_1 vs. FTD_NGF_0 (M-W test, *p* < 0.01) and ADNGF_1 vs. AD_NGF_0 (Mann–Withney test, *p* < 0.05) (Figure 6A).

In addition, we have evaluated the proNGF/NGF ratio, in the subset of samples in which the NGF peak could be reliably measured. As evident from the boxplot in Figure 6B, the ratio proNGF/NGF of FTD samples is significantly lower with respect to AD and SMC samples, while only a trend is noticeable with respect to the control group (Figure 6B). Since the ratio proNGF/NGF can be considered an additional biomarker, we can further confirm the increased ability of distinguishing between FTD and AD by the means of our assay.

### 3.4 Correlation of proNGF and NGF with other biomarkers and clinical records

We have computed the Spearman index in order to evaluate the correlation between proNGF or NGF and the clinically validated biomarkers, as well as the other clinical variables [age, disease duration (in months), years education, time shift (time in months from the first and second symptom), presence of extrapyramidal or motor neuron disease (MND) symptoms, clinical dementia rating (CDR) global score, CDR sum of boxes, FTD-CDR score]. From this analysis, we found that proNGF levels are significantly correlated with pTau levels (Spearman’s rho = −0.31, *p* = 0.048, Supplementary Figure 3A), with the disease duration levels (Spearman’s rho = −0.336, *p* = 0.030, Supplementary Figure 3B), and with NGF levels (Spearman’s rho = 0.702, *p* = 0.00020, Supplementary Figure 3C).

## 4 Discussion

The diagnosis of FTD based only on clinical evaluation criteria is very difficult. There is a recognized need for efficient biomarkers able to increase diagnostic accuracy. The currently validated biomarkers are clinically used for AD diagnosis, or linked to the less represented genetic FTD variants, or related to a generic neuronal damage (Boeve et al., 2022; del Campo et al., 2022; Ulugut and Pijnenburg, 2023).

ProNGF has been suggested as a promising diagnostic biomarker for AD (Counts et al., 2016; Pentz et al., 2020), but almost nothing is known concerning its potential relationship to FTD, also because of the lack of reliable and scalable proNGF assays.

**FIGURE 7:**
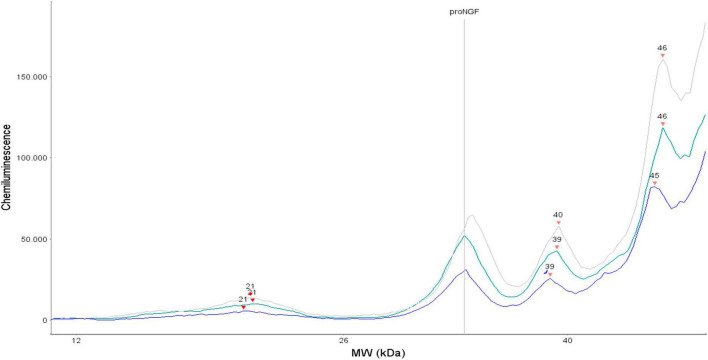
Three representative electropherograms of CSF samples from three different FTD patients.

In this article, we have measured the proNGF levels in 43 CSF samples from FTD living patients by a recently developed and validated immunoassay (Malerba et al., 2015, 2021). We have analyzed the results, and compared them to those recently obtained on 84 AD, 15 SMC, and 13 CTR (Malerba et al., 2021). As indicated in the previous paper (Malerba et al., 2021), we have analyzed only the peak corresponding to the 34 KDa proNGF, the “naked” proNGF form, without post translational modifications. Indeed, also for the FTD samples, three peaks corresponding to proNGF (and in some samples, one peak corresponding to NGF) were identified (Figure 7). We found a significant difference between proNGF levels in FTD samples compared to AD, CTR, and SMC subjects. The 34 kDa proNGF peak area value is lower: ∼71% of AD, and ∼48% of SMC and CTR.

By the means of multivariate models, we have evaluated the accuracy of proNGF in distinguishing FTD from AD, and FTD from controls, in comparison to the clinically validated CSF biomarkers. First of all, we have checked the clinically validated biomarkers in our diagnostic groups and found that their predictivity is in agreement with that reported in literature. Moreover, we found that proNGF is able to discriminate FTD from AD better than Tau and pTau, but, as expected, worse than Aβ42. Remarkably, in the analyzed groups, proNGF appears to be the best biomarker in discriminating FTD from controls.

We have also evaluated the diagnostic performance of proNGF in CSF, if added to the clinical validated biomarkers, and found that it provides more accuracy in the diagnosis, if we compare FTD patients with the control group. Distinguishing FTD patients from non-demented patients is important and not straightforward, due to the fact that FTD clinical symptoms overlap with those of primary psychiatric disorders, in particular in the case of the main phenotypes of bvFTD and PPA (Woolley et al., 2011).

Another promising result obtained by the analysis of CSF from patients was the detection of a mature NGF peak. We demonstrated that also NGF levels (similarly to proNGF levels) were significantly lower in FTD samples compared to CTR and SMC subjects. Moreover, we have added NGF (or NGF + proNGF) as additional predictors to the set of clinically validated biomarkers and found that NGF alone, or NGF + proNGF strongly improved the diagnostic performance in the comparison between FTD and control group.

A further interesting observation is the different percentage of samples having a detectable NGF peak among the diagnostic groups. This indicates that NGF levels are low, below threshold, in a significantly larger proportion of AD samples, with respect to the FTD samples. This observation, together with the finding that proNGF levels are higher in AD versus FTD samples, suggests that a higher proNGF level is not always related to a higher NGF level, as might be expected, but depends on the disease, in a fine tuning of the relative concentrations and distribution of all the actors of the NGF system (proNGF, NGF, and receptors), whose unbalance with respect to the physiological conditions may generate distinct processing patterns and ensuing signaling dysmetabolism, resulting in different diseases. As a practical consequence, we could further stratify FTD and AD populations based on the presence/non-presence of a detectable NGF peak. Moreover, if we analyze the ratio proNGF/NGF in the subset of samples in which the two peaks are present, we found statistically significant differences between AD and FTD.

Taken together, these results have both diagnostic and pathogenesis/therapeutic implications: (1) proNGF (and possibly, NGF and/or their ratio) may provide additional and independent biological information compared to the other biomarkers, providing a further diagnostic accuracy; (2) more globally, these results suggest that a dysmetabolism in the NGF system could be present in FTD and possibly play a role in FTD pathogenesis, as it has been proposed for AD (Capsoni et al., 2011; Tiveron et al., 2013; Pentz et al., 2020). These findings, moreover, might contribute to a more overall view of molecular pathways underlying the pathology and help to discriminate between individual subtypes on the basis of specific molecular features.

As for the evidence pointing to a possible link of the NGF/proNGF system in the pathogenesis of FTD, very little is known from pre-existing literature. On the contrary, NGF dysmetabolism has been widely demonstrated in AD: from the so called “cholinergic theory” (Bartus et al., 1982; Coyle et al., 1983) to the more recent findings (Capsoni et al., 2011; Tiveron et al., 2013; Pentz et al., 2020).

In literature, there are only two studies that correlate NGF/proNGF to FTD. In Belrose et al. (2014), the amount of proNGF in FTD was evaluated by low sensitivity Western blot methods in post mortem parietal cortex of six patients with Pick’s disease (the previous name that indicated bvFTD), six CBS, six PSP, and six controls, finding a statistically significant increase of 34 kDa proNGF in Pick’s disease compared to that in age-matched controls. This increase was not observed in CBS or PSP. Mature NGF was not detected. Another study (Shen et al., 2018) reported an increased expression of proNGF and of the neurotrophin receptor p75NTR, both mRNA and proteins, in the postmortem brains of 10 FTLD tau subjects (compared to 10 controls), as well as in the brain of P301L human tau transgenic mice. A direct link between tau phosphorylation and proNGF via p75NTR in cultured hippocampal neurons from transgenic mice was observed. Moreover, the block of p75NTR signaling or a decrease of p75NTR expression attenuated both the proNGF-induced tau phosphorylation and the behavioral cognitive deficits, suggesting that the proNGF/p75NTR pathway plays a role in tau phosphorylation. Possibly, this might extend to a role of the proNGF/p75NTR pathway in the pathogenesis of FTLD-tau. In keeping with this, from our analysis, a direct correlation between proNGF and pTau levels in CSF from human living patients was found. This evidence points to a new and direct mechanistic link between proNGF processing and signaling and tau pathology. Thus, the hypothesis of a mechanism linking the NGF/proNGF system and the tau pathology would not be exclusively related to the cholinergic system, nor to Aβ processing pathology. Tau pathology, that highly correlated to cognitive decline with respect to Aβ (Giannakopoulos et al., 2003; Chen et al., 2023), is shared by AD and FTD.

Emerging evidences showed a crucial involvement of neuroinflammation across the whole spectrum of FTD, (Yoshiyama et al., 2007; Lant et al., 2014; Bright et al., 2019; Pottier et al., 2019; Woollacott et al., 2020; Chen et al., 2023). Similarly, the prominent role of neuroinflammation in the pathogenesis of AD is largely recognized (Kitazawa et al., 2004; DiSabato et al., 2016; Tischer et al., 2016; Leng and Edison, 2021). On the other hand, NGF and proNGF regulate immune response both in periphery (Williams et al., 2015) and in CNS (De Simone et al., 2007; Capsoni et al., 2011; D’Onofrio et al., 2011; Rizzi et al., 2018; Lisi et al., 2022). Indeed, proNGF is expressed by murine and human astrocytes (Pedraza et al., 2005; Volosin et al., 2006, 2008; Domeniconi et al., 2007) and an increase in proNGF expression and secretion was reported in response to several cases of insults (Volosin et al., 2006; Domeniconi et al., 2007; Cheng et al., 2020), while NGF acts on microglia, steering it toward a neuroprotective and anti-inflammatory phenotype (Rizzi et al., 2018; Lisi et al., 2022).

In this article, we have demonstrated, though indirectly, that in human living patients NGF dysmetabolism is related to tau pathology [so far this connection was demonstrated only in mouse models (Shen et al., 2018)] and hypothesized that this correlation is linked to neuroinflammation process on which NGF system action is relevant.

In this article and in Malerba et al. (2021), proNGF was measured in the peripheral CSF of living patients on a relatively large cohort. On the contrary, in the literature, measurements of proNGF were reported in post mortem tissues on small sample size: in AD brain (Fahnestock et al., 2001; Counts and Mufson, 2005; Al-Shawi et al., 2007; Fahnestock and Shekari, 2019; Pentz et al., 2020), in FTD brain (Belrose et al., 2014; Shen et al., 2018), and only one report in ventricular AD CSF (Counts et al., 2016), by using the low sensitivity Western blot, without calibration curve, finding an increased amount of proNGF in patients with neurodegenerative diseases with respect to non-demented subjects. In our results, the decreased level of the unmodified “naked form” of proNGF in peripheral CSF from AD living patients might appear discrepant to previous literature. This issue was largely discussed in Malerba et al. (2021). Here we report a summary of the possible explanation for this apparent discrepancy.

First of all, proNGF levels in peripheral CSF samples from living patients and controls were never investigated before. Moreover, the fact that the amount of proNGF is increased in the post mortem brain and decreased in the peripheral CSF of living patients could be explained by two hypotheses: (a) a differential partition effect between brain and CSF, similar to that described for Aβ42 brain versus CSF levels, due to trapping of proNGF by binding molecules in the diseased brain, or to a proNGF aggregation process in the brain. In fact, based on the fact that proNGF and NGF reciprocally interfere in the immunoassays (Malerba et al., 2016), we suggested the possible formation of NGF/proNGF supramolecular structures, similar to the described NGF dimer of dimers (Covaceuszach et al., 2015); (b) the presence of pathological post-translational modifications of proNGF, described by us and other authors (Pedraza et al., 2005; Kichev et al., 2009; Malerba et al., 2021). Indeed, we have identified three molecular forms of proNGF, but we have analyzed, due to a technical limitation (largely described in Malerba et al., 2021), only the proNGF peak having the same MW of recombinant proNGF (also claimed in the paragraph “limitation of the study”). We are currently working to optimize our assay in order to be able to analyze the different proNGF forms and their possible interconversion, with the aim of (i) adding knowledge about the biological significance of the different proNGF forms, and (ii) eventually providing other measurable biomarkers. Despite this limitation, the identification of a new candidate biomarker for AD and FTD was reached.

With the aim to improve the clinical diagnosis of FTD, we can hypothesize to set up a complete panel of biomarkers including proNGF and NGF to other fluids or imaging biomarkers related to neuroinflammation (such as for instance in Malpetti et al., 2023) or to NGF metabolism.

On the other hand, the involvement of NGF system in tauopathies should be deeply investigated in order to better understand the molecular mechanism of the FTD pathogenesis.

The results have also a therapeutic implication, in that the possible link between NGF dysmetabolism and FTD pathology warrants the exploration of strategies aimed at re-establishing the homeostatic equilibrium in the NGF/proNGF/p75NTR signaling system.

### 4.1 Limitations of the study

The main limitation of the study to consider is the sample size, which could have challenged the reliability of the more complex models; however, the sample size was adequate considering that FTLD-associated disorders are rare and heterogeneous conditions. Obviously, this limitation impairs the possibility to obtain adequately powered statistical comparisons between the subgroups that were analyzed (not shown).

Concerning diagnostic models, though we are aware that continuous predictors retain more information, the sample size of the study population did not allow us to really exploit the continuous domain for a robust estimate of the thresholds. Moreover, class-prediction models based on real continuous values may also be overfitted, again resulting in unreliable predictions, while we believe that binary predictors may guarantee more robust estimates, though likely losing some power in correctly detecting the diagnosis.

Another point is the fact that we are able to measure only the “naked” proNGF form at the molecular weight of 34 KDa, and not the two higher molecular weight forms, holding post translational modification.

As previously mentioned in the manuscript, a careful and complete validation of the assay was performed for proNGF peak, but not for NGF peaks, due to technical limitations (Malerba et al., 2021). For these reasons, a calibration curve for NGF was not performed. We have demonstrated that NGF, NGF relative percentage, or NGF/proNGF ratio might be considered candidate biomarkers for the mentioned neurodegenerative diseases, but an optimization of the assay for NGF, overcoming technical limitations, must be carried out before undertaking the NGF biomarker validation.

## Data availability statement

The raw data supporting the conclusions of this article will be made available by the authors, without undue reservation.

## Ethics statement

The studies involving humans were approved by the Center for Neurodegenerative Diseases and the Aging Brain of the University of Study of Bari “Aldo Moro” at Pia Fondazione “Card. Panico” Hospital (Tricase). The studies were conducted in accordance with the local legislation and institutional requirements. The human samples used in this study were acquired from primarily isolated as part of your previous study for which ethical approval was obtained. Written informed consent for participation was not required from the participants or the participants’ legal guardians/next of kin in accordance with the national legislation and institutional requirements.

## Author contributions

FM: Conceptualization, Data curation, Funding acquisition, Investigation, Methodology, Supervision, Writing – original draft, Writing – review & editing. RF: Investigation, Writing – original draft. IA: Formal analysis, Methodology, Writing – original draft, Writing – review & editing. CZ: Data curation, Writing – original draft. MD’A: Data curation, Writing – original draft. GL: Data curation, Formal analysis, Funding acquisition, Methodology, Writing – original draft. AC: Funding acquisition, Investigation, Supervision, Writing – original draft, Writing – review & editing.
