# Supplementary material for: Cerebrospinal fluid level of proNGF as potential diagnostic biomarker in patients with frontotemporal dementia
Source: Front Aging Neurosci. 2024 Jan 25;15:1298307. doi: 10.3389/fnagi.2023.1298307 (PMC10850263; doi:10.3389/fnagi.2023.1298307)
Supplement: Supplementary file 1 [file Data_Sheet_1.docx]

Supplementary Material

# Supplementary Figures

**Figure S1.**

Statistical analysis of Tau, Aβ_42_ and pTau in the CSF samples

A


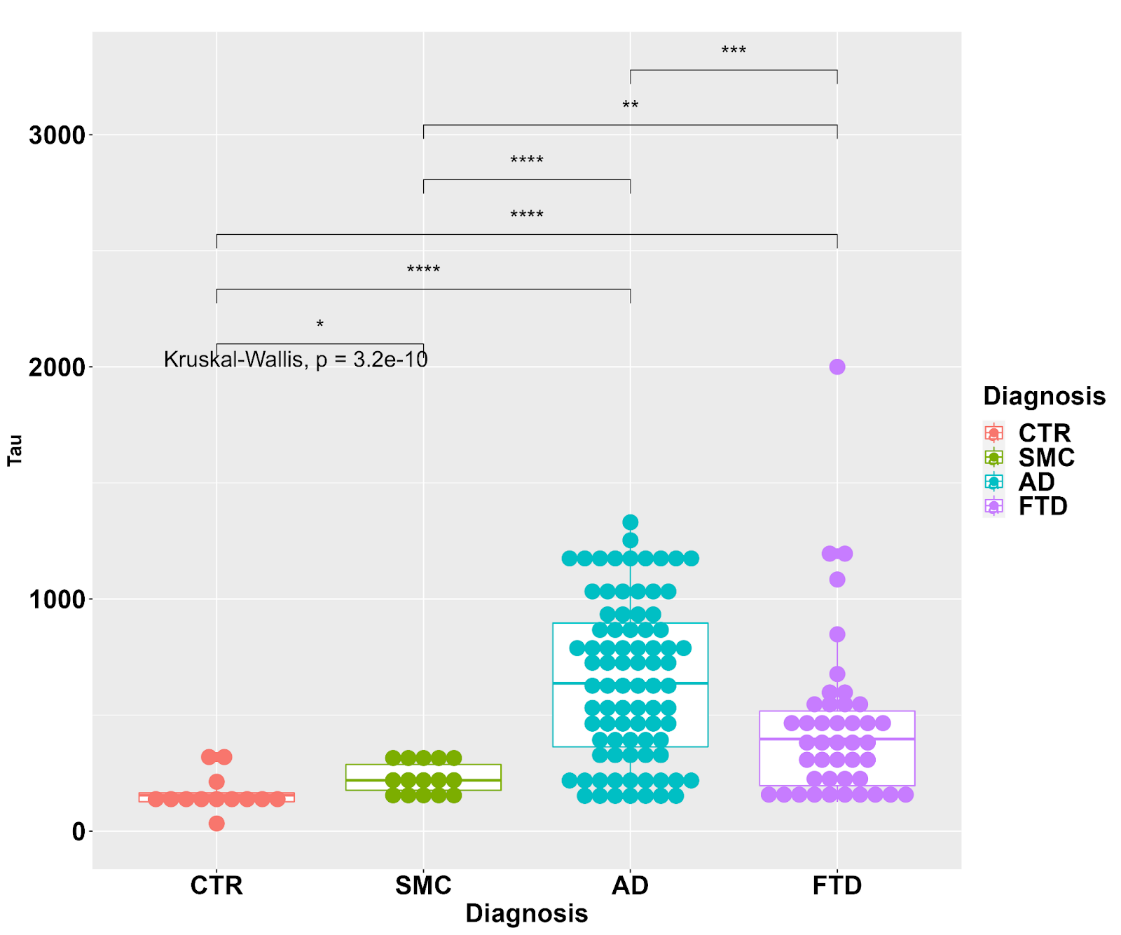


B


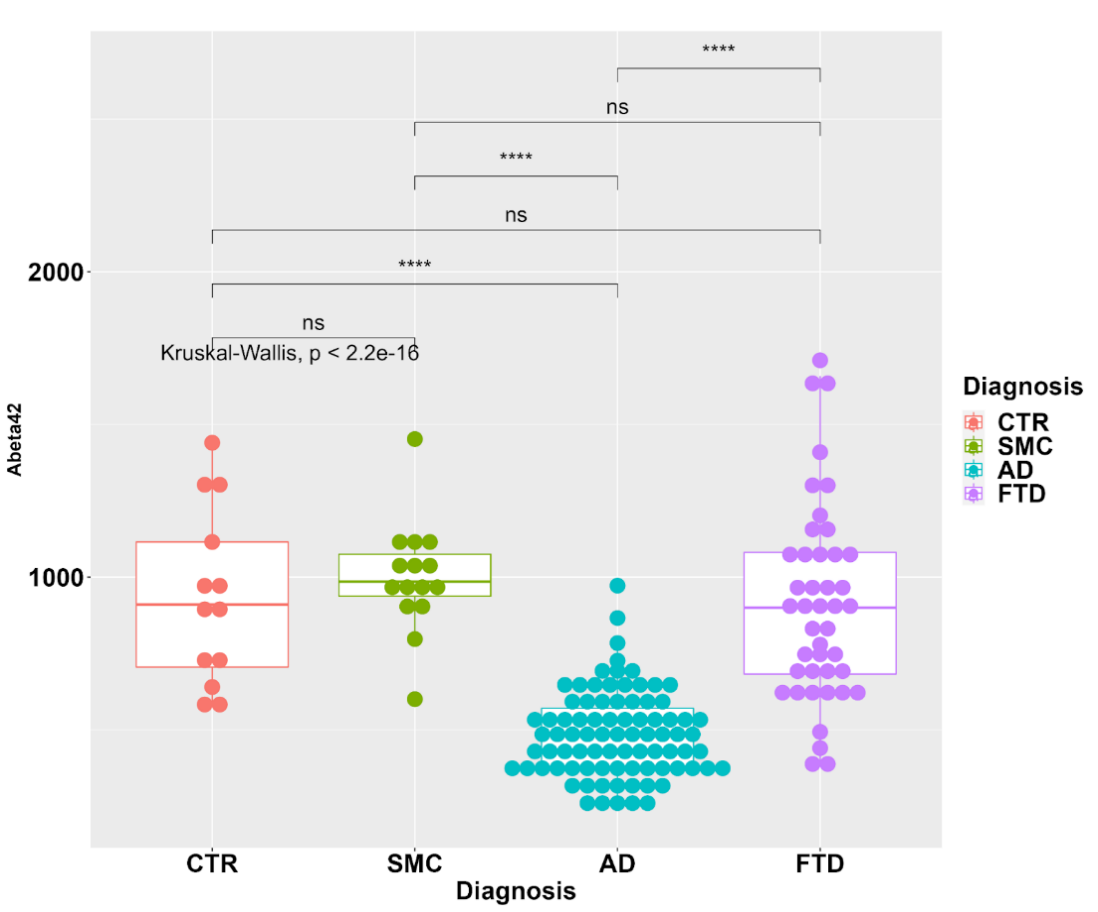


C


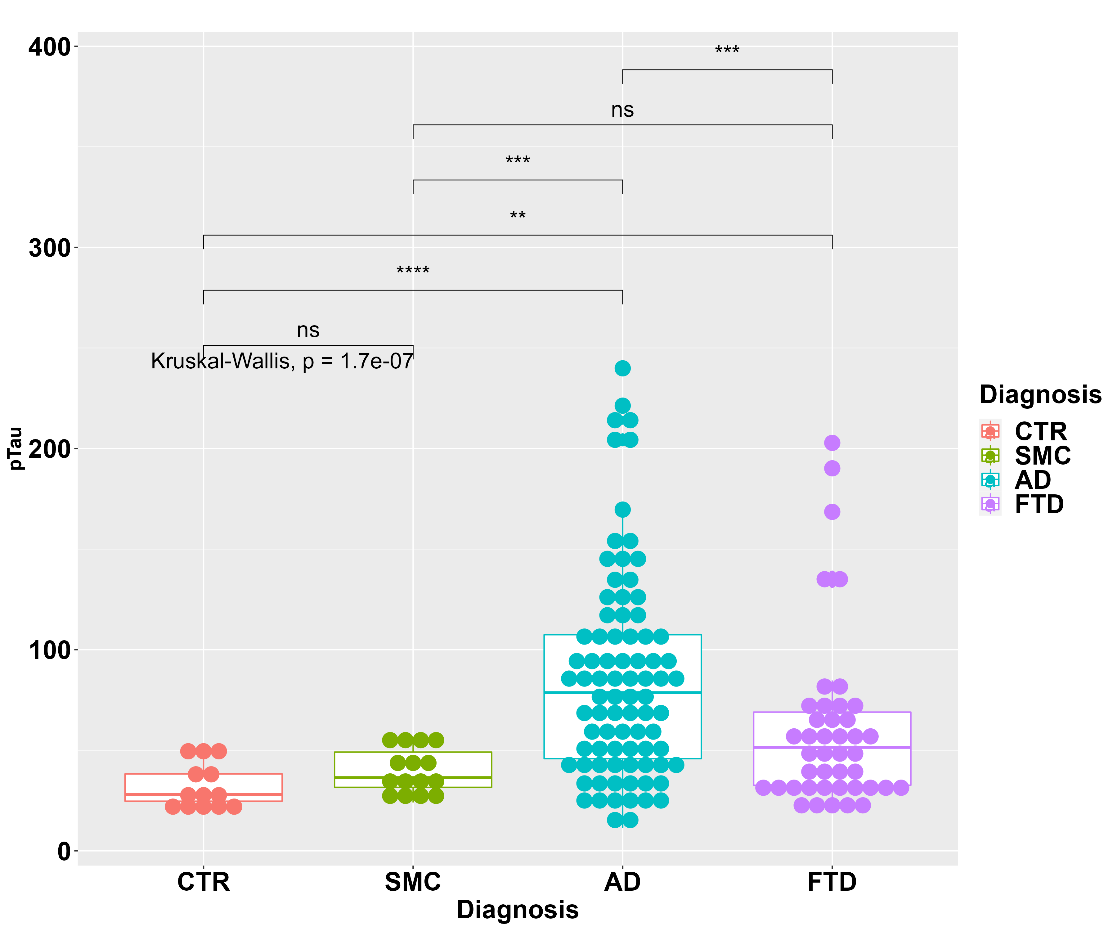


**Figure S1.**

Statistical analysis of Tau, Aβ_42_ and pTau in the CSF samples. Boxplot with dotplot: measure of Tau (A), Aβ_42_ (B) and pTau (C) in the diagnostic groups.

**Figure S2.** ROC curves to show the diagnostic performance of multivariate models comparing FTD vs AD


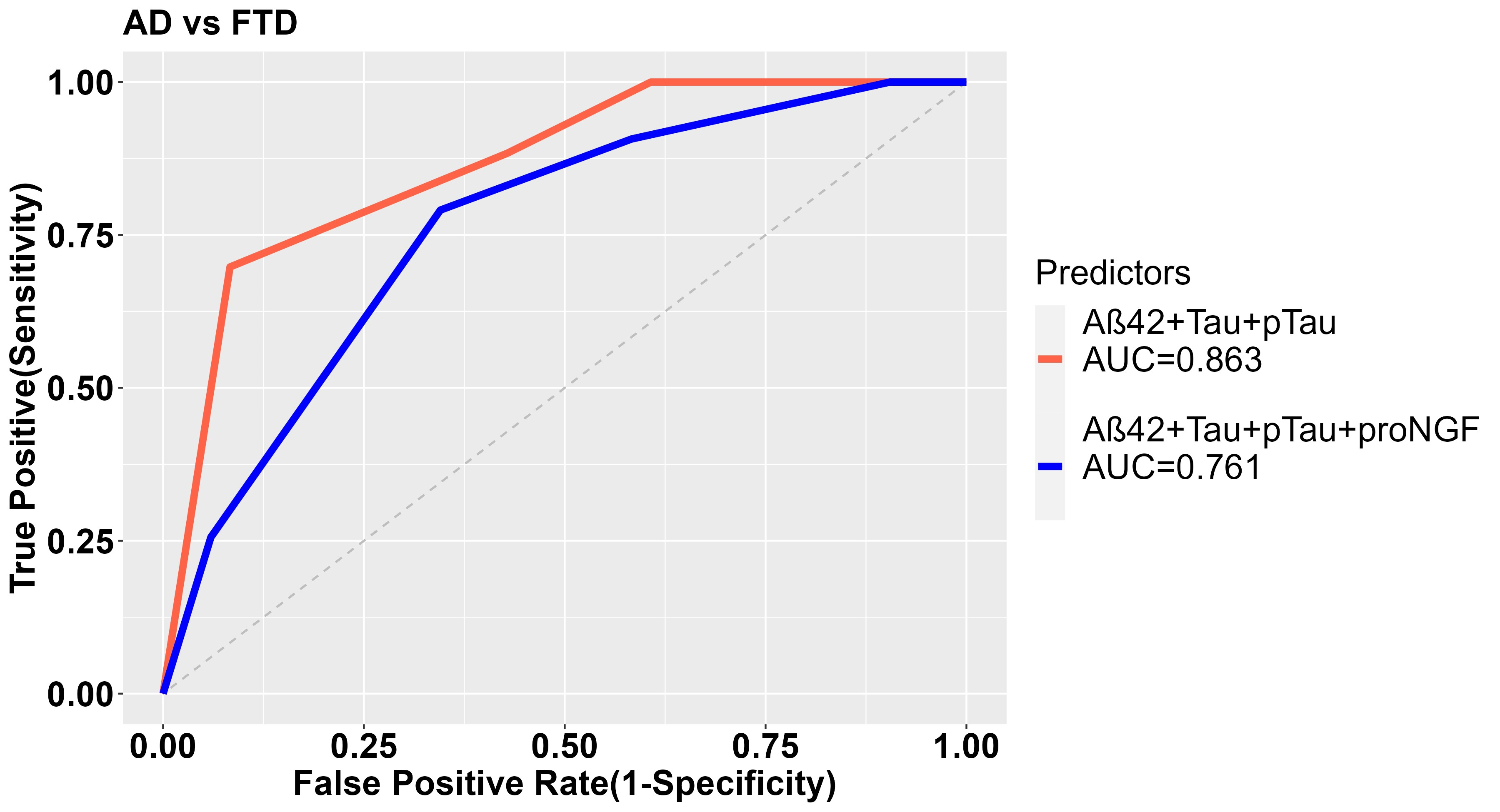


**Figure S2.**

ROC curves showing the diagnostic performance of multivariate models comparing FTD vs AD. The model without proNGF (diagnosis ~ Aβ42 + Tau + pTau), is compared to the same model with proNGF as further predictor (diagnosis ~ Aβ42 + Tau + pTau + proNGF).

**Figure S3** Scatter plot of proNGF levels vs pTau (A), disease duration (B), NGF (C).

**A**


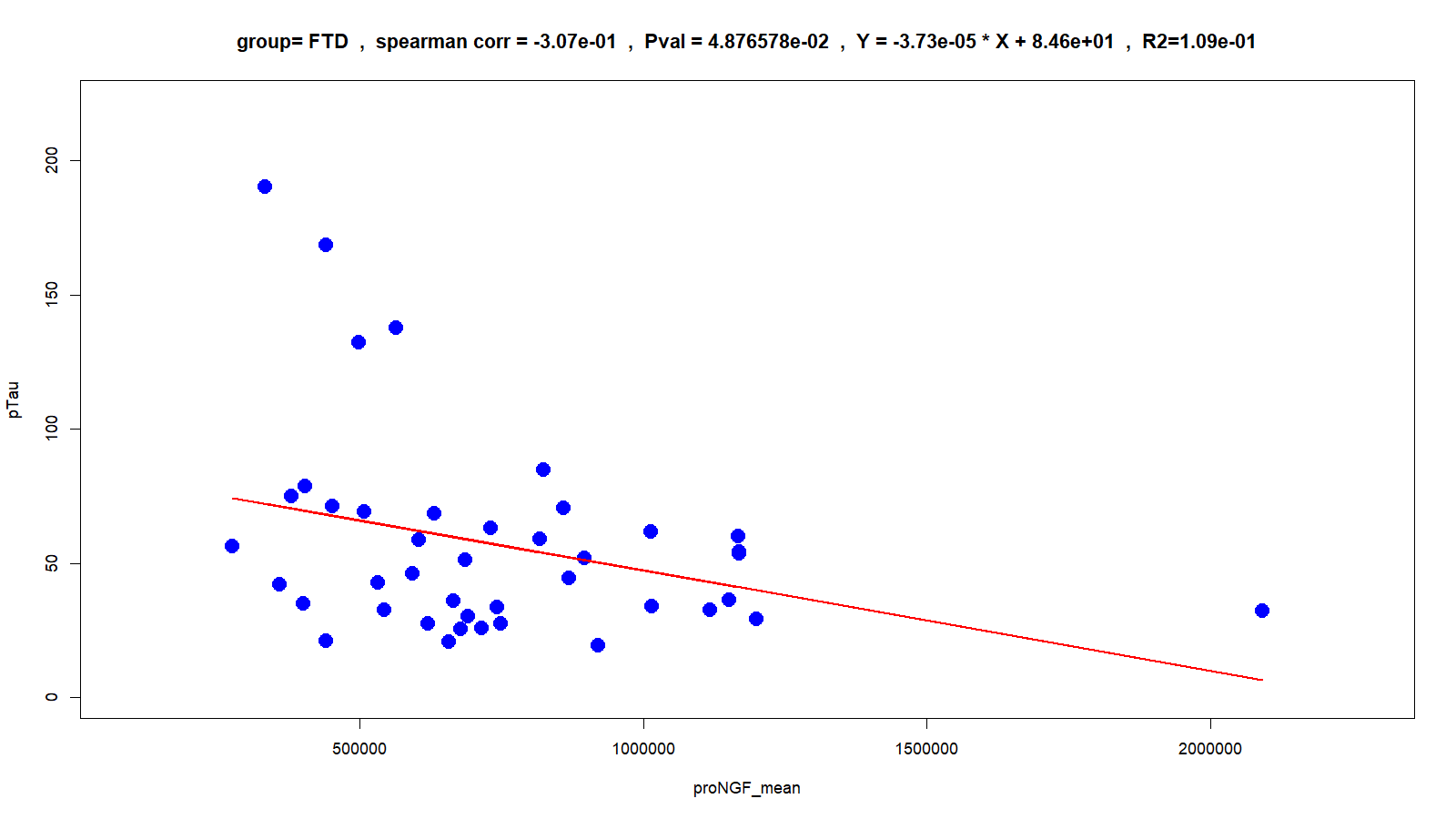


B


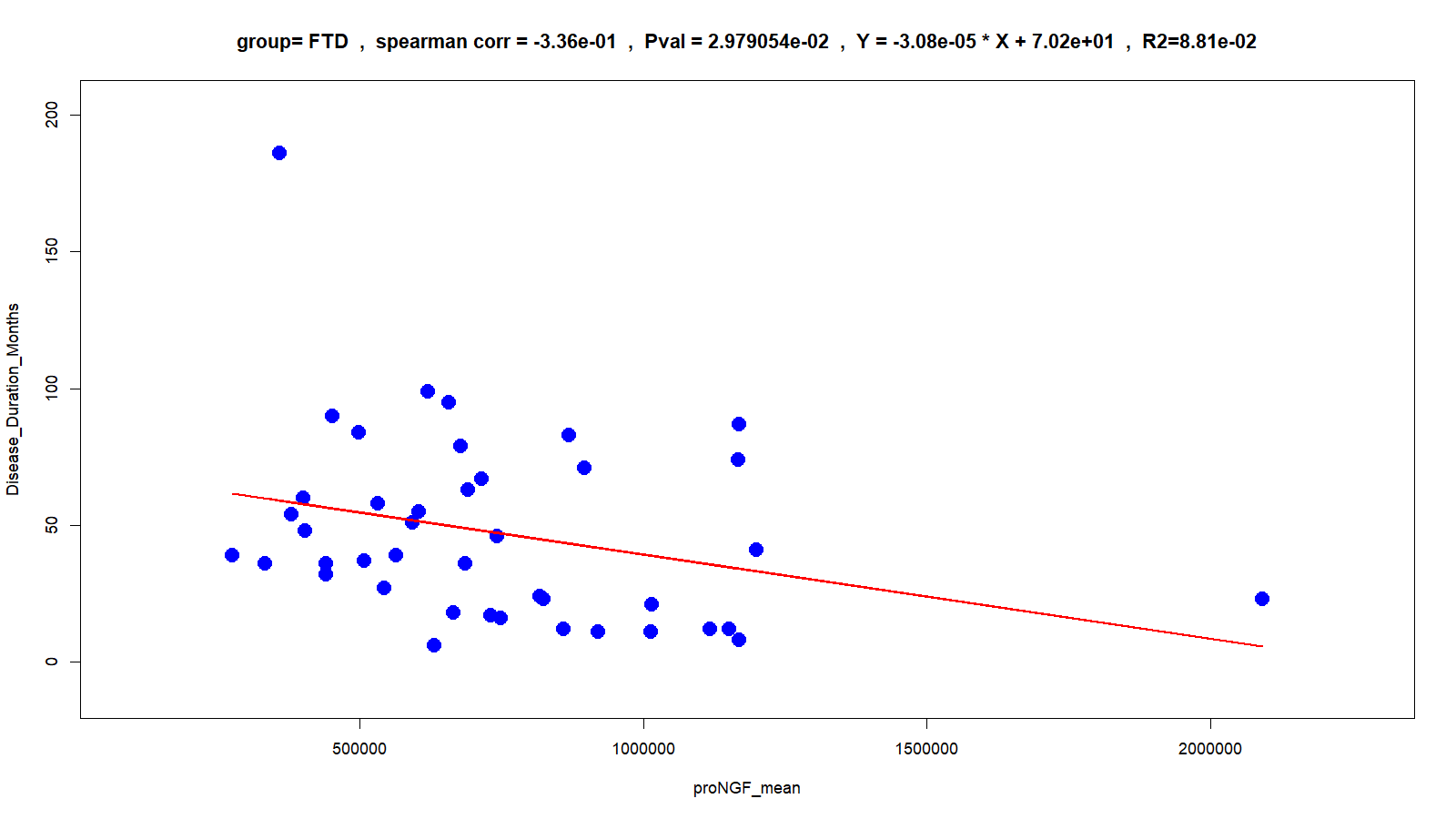


**C**

**
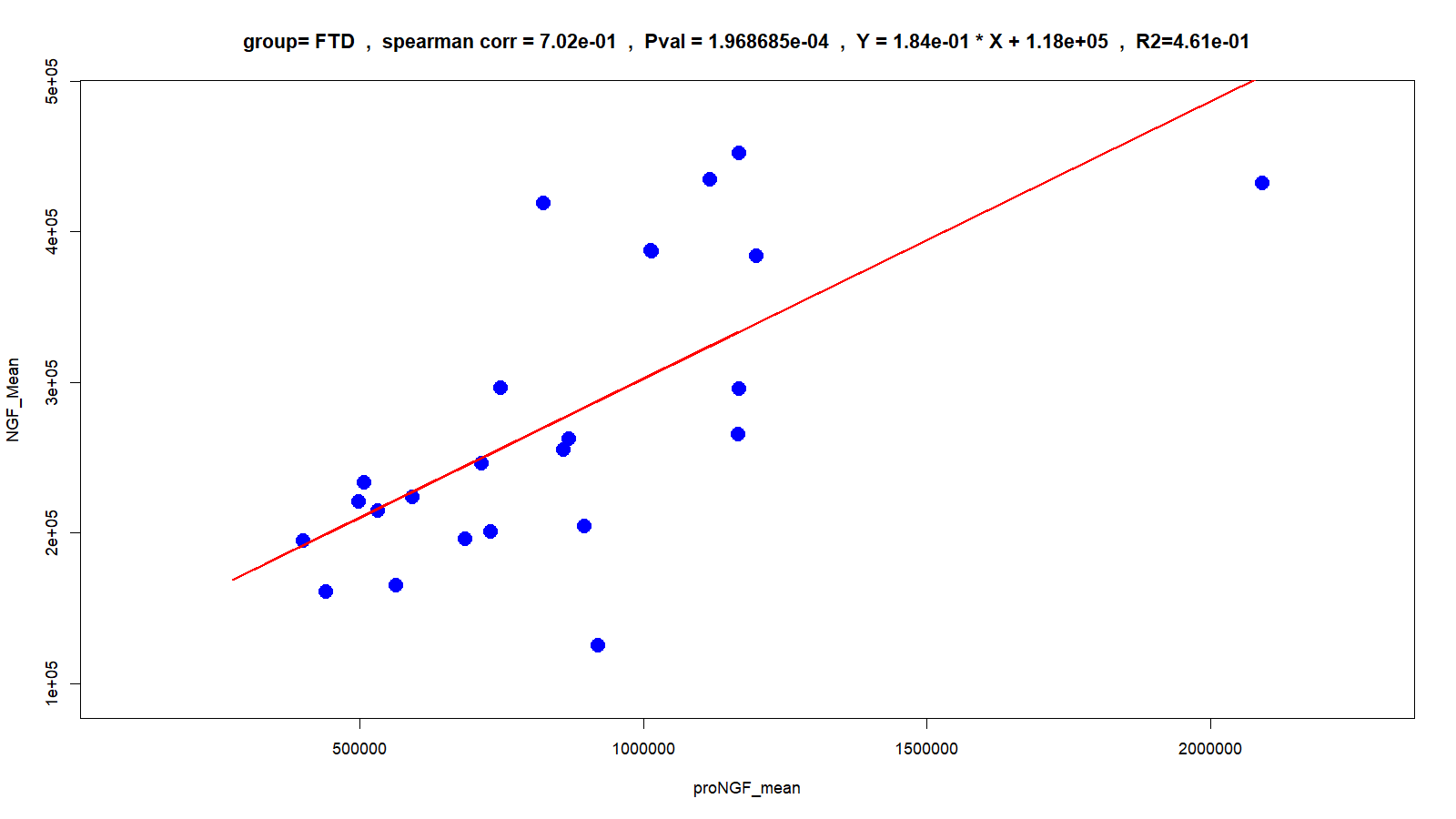
**

**Figure S3.**

Scatter plot of proNGF levels vs pTau (A), disease duration (B), NGF (C). The correlation is evaluated by the Spearman index. The linear regression line is plot in red. The p-value corresponds to the Spearman index. ProNGF in significantly inversely correlated to disease duration and pTau, but positively to NGF.
